# Postnatal Administration of Allopregnanolone Modifies Glutamate Release but Not BDNF Content in Striatum Samples of Rats Prenatally Exposed to Ethanol

**DOI:** 10.1155/2015/734367

**Published:** 2015-02-22

**Authors:** Roberto Yunes, Cecilia R. Estrella, Sebastián García, Hernán E. Lara, Ricardo Cabrera

**Affiliations:** ^1^Instituto de Investigaciones Biomédicas (INBIOMED-IMBECU-CONICET), Facultad de Ciencias de la Salud, Universidad de Mendoza, Paseo Dr. Emilio Descotte 720, 5500 Mendoza, Argentina; ^2^Área de Farmacología, Facultad de Ciencias Médicas, Universidad Nacional de Cuyo, Avenidad del Libertador 80, 5500 Mendoza, Argentina; ^3^Laboratorio de Neurobioquímica, Facultad de Ciencias Químicas y Farmacéuticas, Universidad de Chile, Calle Sergio Livingstone Polhammer 1007, 8380492 Santiago de Chile, Chile

## Abstract

Ethanol consumption during pregnancy may induce profound changes in fetal CNS development. We postulate that some of the effects of ethanol on striatal glutamatergic transmission and neurotrophin expression could be modulated by allopregnanolone, a neurosteroid modulator of GABA_A_ receptor activity. We describe the acute pharmacological effect of allopregnanolone (65 *μ*g/kg, s.c.) administered to juvenile male rats (day 21 of age) on the corticostriatal glutamatergic pathway, in both control and prenatally ethanol-exposed rats (two ip injections of 2.9 g/kg in 24% v/v saline solution on gestational day 8). Prenatal ethanol administration decreased the K^+^-induced release of glutamate regarding the control group. Interestingly, this effect was reverted by allopregnanolone. Regarding BDNF, allopregnanolone decreases the content of this neurotrophic factor in the striatum of control groups. However, both ethanol alone and ethanol plus allopregnanolone treated animals did not show any change regarding control values. We suggest that prenatal ethanol exposure may produce an alteration of GABA_A_ receptors which blocks the GABA agonist-like effect of allopregnanolone on rapid glutamate release, thus disturbing normal neural transmission. Furthermore, the reciprocal interactions found between GABAergic neurosteroids and BDNF could underlie mechanisms operating during the neuronal plasticity of fetal development.

## 1. Introduction

Maternal alcohol consumption during pregnancy induces profound changes in the development of the central nervous system (CNS) in offspring [1]. Although the most critical birth defect associated with alcohol consumption is fetal alcohol syndrome (FAS) [2], many children who were prenatally exposed to alcohol do not present the morphological brain changes characteristically seen in FAS, yet they may still exhibit many of the mental dysfunctions induced by ethanol [3]. Problems characteristically associated with this pathology include impaired cognitive and motor skills, with a reduction in general intellectual functioning, deficits in verbal learning, spatial memory, and reasoning [4].

The basal ganglia appear to be sensitive to prenatal ethanol exposure, as they are clearly smaller in children that suffer from FAS [1]. The corpus striatum, a component of the basal ganglia, is a critical brain structure involved in motor, perceptual, and cognitive skills [5, 6]. It is innervated by excitatory glutamatergic and inhibitory GABAergic neurons. The function of these nerve terminals is finely modulated by neurotransmitters and by neurotrophic factors acting on their specific receptors, the expression of which is altered by prenatal exposure to ethanol [7, 8]. Although the most common way to stimulate the receptors for glutamate and GABA is the ligands themselves, an interesting kind of molecules called neurosteroids may act as modulators of ion channels-coupled receptors and thereby also mediate neuronal responses [9, 10].

Recently, neurosteroids have received much attention as neuroprotective agents against different injuries affecting the CNS [11]. These effects could be modulated, among others, via neuromodulation of GABAergic transmission and less directly by modifying neurotrophin expression, a notion supported by the fact that inducible neurotrophins are downregulated by GABA activity [12]. Recent research shows that allopregnanolone (3*α*-hydroxy-5*α*-pregnan-20-one), a potent positive modulator of GABA_A_ and glutamate receptors activity [13], brain concentration is particularly affected by ethanol administrations in rats [14]. Also, nerve growth factor (NGF) and brain-derived neurotrophic factor (BDNF), members of the neurotrophin family, protect neurons from insults and regulate synaptic transmission in the corpus striatum [15, 16].

Previous reports show that acute ethanol intoxication of rat embryos on gestational day 8 produces postnatal impairments in the neuronal function [17, 18]. In this study we work with a similar experimental paradigm, ethanol intoxication of rat embryos on gestational day 8, in order to study potential modifications of glutamatergic and GABAergic neurotransmission. We postulate that the neurosteroid allopregnanolone administered on postnatal day 21 can possibly override the damaging effect of ethanol by (1) interaction of the neurosteroid allopregnanolone with glutamatergic and GABAergic systems in striatum and (2) modifications of expression of the neurotrophin BDNF. Both results allow us to postulate the use of neurosteroids as potentially useful molecules in neuroregeneration after brain damage induced by prenatal ethanol exposure.

## 2. Materials and Methods

### 2.1. Reagents

The used reagents were as follows: ethanol (Biofarma); allopregnanolone (Sigma, St. Louis, MO, USA); (^3^H)-glutamic acid (^3^H-Glu); (^3^H)-*γ*-aminobutyric acid (^3^H-GABA) (New England Nuclear (Boston, MA, USA)); carbogen (95% O_2_ and 5% CO_2_) Krebs-Ringer bicarbonate glucose buffer (KRB, pH 7.4) or Mg^2+^-free KRB (experiments with ^3^H-Glu); BDNF Emax ImmunoAssay System kits from Promega (Madison, WI, USA).

### 2.2. Animals and Prenatal Exposure to Ethanol

The procedure used to expose rats to ethanol on gestational day 8 (GD8) has been previously described [19]. Adult male and female Sprague-Dawley rats (280–300 g) from our own breeding colony were used. Animals for these experiments were kept and handled according to the Guide for the Care and Use of Laboratory Animals, Institute of Laboratory Animal Research, Commission on Life Sciences, National Research Council, USA [20]. All efforts were made to minimize animal suffering. They were housed under a constant temperature of 22 ± 1°C and a standard light/dark cycle (12 h light schedule lights on at 07:00 a.m.) and controlled environment with food and water* ad libitum*. Samples for vaginal smears were taken daily from 09:00 to 10:00 a.m. Only rats that showed more than two normal 4-5-day consecutive estrus cycles were used for the experiments. On the evening of proestrus, they were housed overnight with male rats. Presence of spermatozoa in vaginal smears the following morning was interpreted as an index of pregnancy and was referred to as gestational day 1 (GD1). On GD8 animals were randomly divided into two groups: one of them received two intraperitoneal (ip) injections of ethanol (2.9 g/kg in 24% v/v saline solution), spaced by an interval of 4 h, and the other one received an identical volume of the saline, also spaced by a 4 h interval (control). Delivery day was considered as postnatal day 1 (P1). All litters were normalized to 10–12 pups/rat and were maintained until postnatal day 21 (P21). On P21 only male pups were selected for the experimental protocol.

### 2.3. Neurosteroid Treatment

On P21, ethanol and control males pups (*n* = 6–9) were s.c. injected with allopregnanolone (65 *μ*g/kg) dissolved in propylene glycol (0.2 mg/mL) or with the same volume of vehicle. Allopregnanolone was initially dissolved in propylene glycol to a concentration of 600 *μ*M. The dose of allopregnanolone used in the experiments (65 *μ*g/kg) was obtained by successive dilution in sterile saline. We selected this dose because it is approximately two hundred times higher than in serum concentration obtained in the night of proestrus in intact female rats [21]. After 2.5 h they were decapitated and the brains were rapidly removed. The corpus striatum was immediately dissected out on ice for release experiments. For neurotrophin content measures, the striatum was removed and stored at −80°C until the day of the measurement procedure.

### 2.4. Release Experiments

Dissected striatal samples from 6–9 rats were sliced at 240 *μ*m with a McIlwain tissue chopper and 6 to 8 slices (experimental tissue) were exposed to 2.79 *μ*M (^3^H)-glutamic acid (^3^H-Glu) (specific activity 44 Ci/mmol) or 940 nM (^3^H)-*γ*-aminobutyric acid (^3^H-GABA) (specific activity 93.20 Ci/mmol) in 2 mL of gassed (95% O_2_ and 5% CO_2_) Krebs-Ringer bicarbonate glucose buffer (KRB, pH 7.4) or Mg^2+^-free KRB (experiments with ^3^H-Glu), for 15 min at 37°C. Slices were transferred to perfusion chambers and superfused at a flow rate of 0.7 mL/min with KRB for 30 min to remove nonincorporated neurotransmitter (washing period). After that 5 fractions of samples (2 min each) were collected and considered as basal release. Thereafter, a depolarizing concentration of K^+^ (KCl 28 mM) was introduced by addition in the superfusion system for a 6 min period. Fractions collected during this time were considered as stimulated release. At the end of the experiment, slices were homogenized in 2 mL 0.2 N perchloric acid in order to obtain the total amount of radioactivity loaded by the tissue. Aliquots of 500 *μ*L of the samples and homogenized tissue were mixed with scintillation solution. The radioactivity of collected fractions and tissue samples was determined, and the percentage of stimulus-evoked release of ^3^H-Glu or ^3^H-GABA was calculated as the percentage of increase over baseline after stimulation [22].

### 2.5. ELISA Assays

ELISA assessments for brain-derived neurotrophic factor (BDNF) were performed using BDNF Emax ImmunoAssay System kits from Promega (Madison, WI, USA). Neurotrophin protein content of striatum was obtained from 4-5 rats. Each ELISA 96-well plate was coated with anti-BDNF monoclonal antibody (1 : 1000; 100 *μ*L/well) in carbonate coating buffer (0.05 M sodium bicarbonate/carbonate; pH 9.7). The wells were sealed with a plate sealer and incubated overnight at 4°C. On the following day, the content of the wells was removed and the plates were washed with Tris-buffered saline with Tween (TBST; 20 mM Tris-HCl, pH 7.6; 150 mM NaCl; 0.05% v/v Tween-20). Thereafter they were incubated at room temperature (RT) for 1 h in block and sample buffer (BSB, 200 *μ*L/well). Tissue samples were weighed, homogenized in 1 : 50 Dulbecco's phosphate-buffered saline (DPBS; pH 7.35), and centrifuged for 15 min (13000 rpm). Two columns on the ELISA plates were designated for the neurotrophin standard curve, which ranged from 0 to 500 pg/mL. A 200 *μ*L aliquot of the tissue sample was added to a well and 3 successive 1 : 2 dilutions were made. Samples and standards were incubated for 6 hours, washed, and then incubated overnight at 4°C with anti-NGF or anti-BDNF antibodies. At the end of the procedure, plates were washed and incubated with an anti-rat IgG-HRP conjugate for 2.5 h at RT. After the incubation, TMB one solution (tetramethylbenzidine and the peroxidase substrate) was added to each well, and the plates were shaken for 5–10 min at RT; the reaction was terminated by adding 1 N HCl to the wells. The plates were read at 450 nm using a monochromator microplate reader (Safire 2 Team), and the results were normalized per gram of tissue assayed [23].

### 2.6. Statistical Analyses

Data were expressed as the mean ± SEM and analyzed using a two-way analysis of variance parametric test (ANOVA 2), followed by a post hoc test whenever necessary (Newman-Keuls test). *P* < 0.05 was considered as the minimum criterion for assigning statistical significance.

## 3. Results

### 3.1. The Effect of Allopregnanolone on Striatal ^3^H-Glutamate Uptake and Release in Male Rats Prenatally Exposed to Ethanol

**Figure 1:**
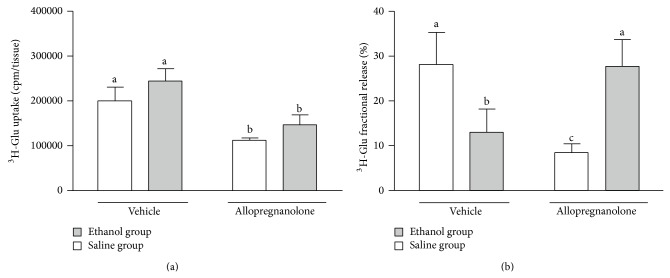
Panel (a): ^3^H-Glu uptake from rat striatal slices; Panel (b): ^3^H-Glu fractional release induced from rat striatal slices by 28 mM K^+^. Control (white bars) or prenatal ethanol-exposed (gray bars) male rats were injected with allopregnanolone (65 *μ*g/kg) or vehicle on P21, 2.5 h before the experiment. Results are expressed as mean ± SEM (*n* = 6–9) (different letters indicate statistically significant differences).

As an index of the activity of the glutamatergic neurons, induced by prenatal ethanol treatment and acute postnatal pharmacological treatment with allopregnanolone we measured changes in the uptake and release of glutamic acid from corpus striatum slices obtained from the animals that received or did not receive prenatal ethanol. No change in striatal ^3^H-Glu uptake was observed after prenatal exposure to ethanol (Figure 1(a)). On the other hand, both control and prenatal ethanol-exposed rats injected s.c. with 5 allopregnanolone decreased the ^3^H-Glu uptake (*P* < 0.05) in relation to the vehicle injection alone (Figure 1(a)). A clear decrease of ^3^H-Glu release was found in response to the depolarizing stimulus in the prenatal ethanol-exposed rats (Figure 1(b)). The injection of allopregnanolone significantly decreased the stimulus-evoked release of ^3^H-Glu in control rats (*P* < 0.01). Interestingly, the opposite trend is observed in the prenatal ethanol-exposed rats injected with allopregnanolone (Figure 1(b)).

### 3.2. The Effect of Allopregnanolone on Striatal ^3^H-GABA Uptake and Release in Male Rats Prenatally Exposed to Ethanol

As an index of the activity of the GABAergic neurons, induced by prenatal ethanol treatment and acute postnatal pharmacological treatment with allopregnanolone, we measured changes in the uptake and release of GABA from corpus striatum slices obtained from the animals that received or did not receive prenatal ethanol. ^3^H-GABA uptake did not change under different experimental conditions. Also, no changes in striatal ^3^H-GABA stimulus-evoked release were found after exposure to ethanol or after acute allopregnanolone administration to the rats.

### 3.3. The Effect of Allopregnanolone on BDNF Concentrations in Prenatally Ethanol-Exposed Rat Brain

**Figure 2:**
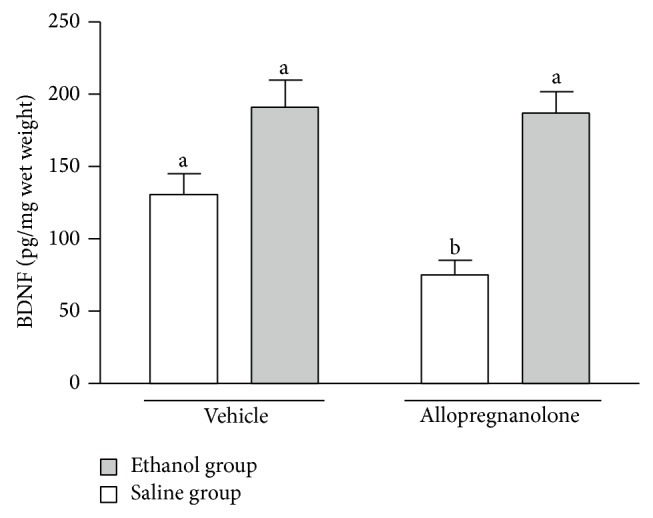
Effect of allopregnanolone (65 ug/kg) on BDNF content in P21 rat striatum. Control (white bars) or prenatal ethanol-exposed (gray bars) male rats were injected with allopregnanolone or vehicle on P21, 2.5 h before the experiment. Results are expressed as mean ± SEM (*n* = 4-5) (different letters indicate statistically significant differences).

As an index of neurotrophin participation in the changes of the activity of glutamatergic neurons induced by prenatal ethanol and by allopregnanolone administration, we measured the changes in BDNF protein content in brain tissues that are known to have the receptors for these neurotrophins. Allopregnanolone significantly decreased BDNF levels in striatum of control rats (*P* < 0.05) but not in ethanol-exposed rats (Figure 2).

## 4. Discussion

Herein we report that in control rats the administration of allopregnanolone in a single subcutaneous dose decreases the K^+^-induced release of glutamate from corpus striatum slices* in vitro* (Figure 1(b)) and decreases the BDNF protein content in this structure (Figure 2). Since our subjects were studied just 2.5 hours after injection of allopregnanolone, we think this could represent a rapid agonistic effect on GABA_A_ membrane receptors coupled to ionic channels [13, 24]. The uptake of glutamate was also decreased by allopregnanolone in the striatum of control animals (Figure 1). Both glutamate uptake and release decrement by allopregnanolone administration, as well as the decrease in the BDNF content in the striatum, could be due to an inhibitory effect of the neurosteroid on the glutamatergic corticostriatal pathway [25–28]. If so, it is possible to speculate that the decrease in the striatal content of the neurotrophin could be a consequence of GABAergic activation by allopregnanolone in control animals.

Prenatal administration of ethanol shows that acute intoxication during GD8 induced long-term changes in the CNS of the offspring [19]. In this work a similar procedure was effective in blocking allopregnanolone action on both glutamate release and BDNF lowering (Figures 1(b) and 2). Allopregnanolone also failed to inhibit the ^3^H-GABA release from striatal interneuron, which expresses GABA_A_ receptors, in ethanol-treated rats. In addition to the mechanism described above, this observation could be explained by a functional alteration of GABA_A_ receptors induced by prenatal ethanol treatment [8]. This suggestion is based on behavioral data pointing out that prenatal exposure to ethanol can alter the GABA_A_ sensitivity to the modulatory effects of allopregnanolone [8, 29, 30]. Furthermore, it was reported that prenatal exposure to ethanol can produce long-lasting alterations in the neuromodulatory influences on GABA_A_ receptor-mediated transmission, as a consequence of either differential GABA_A_ receptor subunit expression or receptor uncoupling [31, 32]. Thus, ethanol-induced alterations in GABA_A_ subunits expressed in the prefrontal cortex may play a role in the observed modulatory responses to allopregnanolone. It is possible that both of these mechanisms, one as a preferential action of allopregnanolone at cortical level and the other affecting the expression of GABA_A_ receptor subunits, may contribute to allopregnanolone effects on glutamate release in ethanol-exposed rats. Since allopregnanolone did not modify striatal BDNF content in these rats, it might also contribute to restoring the excitatory tone toward normal values. Whether or not these modifications are persistent is an issue to be elucidated in the near future.

Altogether the data reported here show that a single dose of allopregnanolone administrated to control rats inhibited excitatory neurotransmission and decreased BDNF protein content at corpus striatum. Ethanol exposure* in utero* may produce alterations in GABA_A_ receptors that could explain the rapid modification in glutamate release and the synthesis and/or secretion of cortical BDNF. Thus, the effects of alcohol on GABA_A_ receptors may induce the trophic and regulatory actions of neurotrophins. Moreover, interactions between endogenous GABAergic-like neurosteroids such as allopregnanolone and certain neurotrophins could underlie mechanisms operating within the brain's developmental plasticity, giving protection of the fetus against insults such as ethanol prenatal exposure.
